# Innovative hybrid molecularly imprinted sensor for the detection of the antioxidant biomarker catalase

**DOI:** 10.1007/s00216-026-06356-x

**Published:** 2026-03-10

**Authors:** Alaa A. Hasseb, N. T. Abdel Ghani, Ola R. Shehab, Rasha M. El Nashar

**Affiliations:** Chemistry Department, Faculty of Science, Cairo University, Giza, 12613 Egypt

**Keywords:** Molecularly imprinted polymers, Silver nanoparticles (AgNPs), Catalase (CAT), Electropolymerization, Biomarkers

## Abstract

In this study, a novel hybrid molecularly imprinted polymer (MIP)-based biomimetic sensor was fabricated, optimized, characterized, and applied for the electrochemical recognition of catalase (CAT), an antioxidant biomarker. A layer-by-layer approach was followed to prepare the hybrid sensor. First, silver nanoparticles (AgNPs) were deposited by chronoamperometry on a bare glassy carbon electrode (GCE) for 300 s, followed by drop-casting of multi-walled carbon nanotubes (MWCNTs). The MIP was then produced by ten voltammetric electropolymerization cycles using *ortho*-aminophenol (*O*AP) as a functional monomer in the presence of catalase as a template on the MWCNT/AgNP/GCE surface at a potential range of −0.2 V to 0.8 V. The fabricated sensor exhibited a rapid response toward CAT within a linear range of 25–500 U/mL and a limit of detection (LOD) of 3.59 U/mL, with no cross-reactivity toward several tested possible biological species. CAT was also successfully detected in biological samples, with recovery percentages ranging from 95.73% to 101.60% and relative standard deviation (RSD) of 0.41–1.52%, which is comparable to the results for a commercial diagnostic reference kit, without the need for complicated sampling procedures. The sensor performance is dependent on catalase molecular recognition rather than reactivity towards its substrate. This feature, in addition to the sensor’s low cost and portability, positions it as a promising candidate for use in point-of-care diagnostics in the medical field.

**Graphical Abstract:**

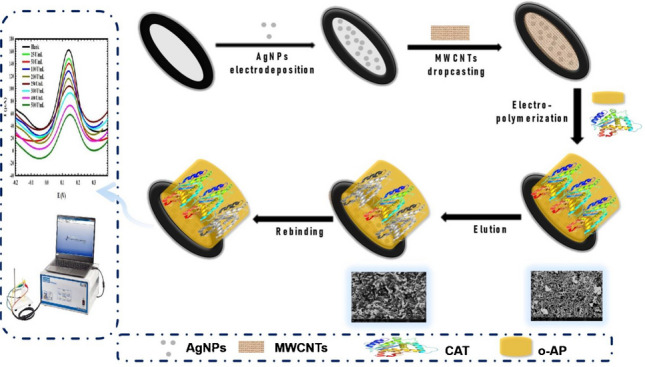

## Introduction

Enzymes control the majority of the chemical reactions that occur in all living organisms, known as biological processes. However, these reactions would not occur at a noticeable rate in the absence of the involved enzymes, leading to the disruption of vital activities, manifested as symptoms and biomarkers produced by different organs [1]. Hydrogen peroxide (H_2_O_2_) is one of the most common oxidative forms in biological systems [2, 3]. The first effective interaction mechanism, H_2_O_2_, is achieved using powerful antioxidant enzymes like catalase, glutathione peroxidase, and superoxide dismutase [4].

Catalase (EC 1.11.1.6), a heme-containing tetrameric enzyme, is recognized as a key antioxidant enzyme, acting to significantly reduce cellular oxidative stress by converting H_2_O_2_ into oxygen and water, thus shielding biomolecules from oxidative damage. CAT is used not only by humans but also for biological processes in all aerobic microorganisms, animals, and plants [5, 6]. CAT is also reported as a promising biomedical tool for preventing or treating respiratory syncytial virus infection [7] and acts as a biomarker in thyroid gland disorders, as indicated by its overexpression. Furthermore, it is also reported as a potent biological indicator for several other severe diseases that are associated with reduced or altered catalase activity, such as Parkinson’s disease [8], type II diabetes mellitus [9], carcinogenic disease [10], COVID-19 [11], Wilson’s disease, hypertension, anemia, several dermatological disorders, bipolar disorder, schizophrenia, Alzheimer’s disease, cardiovascular disorders, and chronic obstructive pulmonary disease [12–16]. Numerous instrumental techniques have been reported for the determination of CAT, including high-performance liquid chromatography [17], spectrophotometry [5, 18, 19], and chemiluminescence [20]. Most of these documented techniques are complicated and costly to execute; the instruments used are either very expensive or not readily adaptable.

Electrochemical sensors are commonly applied as a fast and reliable alternative approach for catalase detection. Examples include electrochemical impedance spectroscopy (EIS) and cyclic voltammetry (CV) applied using a glassy carbon electrode (GCE) modified by multi-walled carbon nanotubes (MWCNTs) and coated in a poly(safranine T) polymer film [13], and flow injection analysis using a GCE modified with electrochemically deposited gold nanoparticles on MWCNTs/chitosan film [21].

Molecularly imprinted polymers (MIPs) represent a class of synthetic polymeric matrices that can be designed and tailored for the extraction or detection of a specific target analyte, depending on the type of application. MIPs are increasingly being explored as potential artificial receptors with the ability to recognize several biological materials [22, 23], including enzymes [24], proteins [25], microRNA (miRNA) [26], viruses [27], and biomarkers [28, 29]. MIPs are used as recognition elements in electrochemical sensors because of their unique benefits, including speed, affordability, and accurate and selective outcomes as compared to other routine procedures for biological marker detection [25, 30].

Literature screening of previously reported methods for CAT detection revealed that none involved the use of such a targeted class of artificial receptors; thus, in this work, molecular imprinting technology is reported for the first time.

Electropolymerized MIPs produce intriguing electrochemically selective materials that mimic the biological antibody–antigen systems [31] directly on the surface of the working electrode. Also, the integration of MIPs with nanomaterials or epitope imprinting can help to enhance the charge transfer and signal transduction taking place at the sensor’s interface, which has a direct impact on the sensitivity toward the target template [32, 33].

The use of electropolymerization for imprinting enables polymer synthesis from an aqueous solution under optimal conditions, and thus it overcomes several drawbacks of traditional bulk polymerization [34]. Consequently, electropolymerization is frequently employed for imprinting many biological macromolecules to avoid the possible destruction or denaturation that may be caused by organic solvents in the case of conventional free radical initiation or thermal initiation polymerization [24, 35].

Silver nanoparticles (AgNPs) have attracted considerable attention because of their outstanding physicochemical characteristics, with widespread use in biomedical research, catalysis, and sensing. AgNPs can be prepared using different approaches including physical, chemical, and biological synthesis methods [36, 37]. Because of their superior electrical conductivity, AgNPs can transfer electrons between the electrode and analyte quickly, achieving improved detection limits. AgNPs have been reported as promising choices for electrochemical sensor applications [38, 39].

Because of their excellent mechanical, electrical, and thermal conductivity characteristics, MWCNTs have been used in numerous reported electrochemical sensor designs [40, 41]. MWCNTs are an excellent choice for the support material in MIP technology because of their vast surface area, providing a large number homogeneous binding sites [42]. Additionally, many researchers have combined AgNPs and MWCNTs for the synergistic improvement of electrode performance [42].

In the current work, for the first time, a hybrid MIP design is presented for CAT whole-cell recognition using a GCE modified with *O*AP as a functional monomer for targeted recognition of CAT in the presence of a AgNP/MWCNT nanocomposite as an electrical signal enhancer. A solution of 2.5 mM [Fe(CN)_6_]^3−/4−^ (FCN) was used as an electroactive probe to detect the interaction between the specific fabricated binding sites on the sensor’s surface and CAT, without the use of H_2_O_2_ as the substrate. Thus, there is no need to test the activity of the enzyme; rather, only the amount of the enzyme in the sample is determined, which represents an advantage for the proposed sensor relative to previously reported sensors that require testing of CAT activity prior to measurements, and the response toward H_2_O_2_ is the measured signal rather than the content of the enzyme in the matrix. Accordingly, the sensor can be used for the efficient clinical testing of CAT comparable to commercial spectrophotometric enzyme detection kits, without the need for extraction or complicated sampling steps.

## Experimental

### Reagents and materials

All chemicals used in this study were of analytical grade and were used without any further refinement. Bovine liver catalase (EC 1.11.1.6), *O*AP, potassium dihydrogen phosphate, dipotassium hydrogen phosphate, methanol (99%), dimethyl sulfoxide (DMSO, 99%), dimethyl formamide (DMF), sulfuric acid (98%), phosphoric acid (85%), nitric acid (70%), potassium chloride, potassium ferricyanide, potassium ferrocyanide trihydrate, urease, glucose, uric acid, creatine, and xanthine were purchased from Sigma-Aldrich (Germany). MWCNTs with a diameter range of 10–20 nm and length of 5–15 μm were purchased from Tokyo Chemical Industries, Japan. Ultrapure (Milli-Q) water purified in a PURELAB UHQ (ELGA, UK) was used for preparations throughout this study.

The commercial spectrophotometric kit for the calorimetric detection of catalase was purchased from Biodiagnostic (Egypt) and consists of phosphate-buffered saline (PBS, pH 7.0), H_2_O_2_, chromogen-inhibitor, peroxidase enzyme, and 4-aminoantipyrine preservative.

The method involves the use of a known amount of H_2_O_2_ to react with catalase, and the reaction is stopped after 1 min using a catalase inhibitor.

$$2{\mathrm H}_2{\mathrm O}_2\overset{\mathbf{CAT}}{\mathit\rightarrow}2{\mathrm H}_2{\mathrm O}_2+{\mathrm O}_2$$

Horseradish peroxidase (HRP) causes the remaining H_2_O_2_ to react with 4-aminophenazone (AAP) and 3,5-dichloro-2-hydroxybenzene sulfonic acid (DHBS) to create a chromophore whose color intensity is inversely proportional to the quantity of catalase present in the original sample.

$$2{\mathrm H}_2{\mathrm O}_2+\mathrm{DHBS}+\mathrm{AAP}\xrightarrow{\mathbf{HRP}}\mathrm{Quinoneimine}\;\mathrm{Dye}+4{\mathrm H}_2\mathrm O$$

### Apparatus

All electrochemical measurements were carried out using a 620E electrochemical analyzer (CH Instruments, Bee Cave, TX, USA) in a three-electrode cell, composed of a 3-mm CHI104 GCE (CH Instruments Inc., USA), a 1-mm platinum wire, and an external Ag/AgCl as a working, counter, and reference electrode, respectively. EIS was performed using a PalmSens 4 potentiostat/galvanostat (PalmSens BV, Houten, Netherlands), and the surface was analyzed using scanning electron microscopy (SEM) (FEI Quanta 250 FEG high-resolution SEM) at Desert Research Studies Center. The surface morphology was characterized by atomic force microscopy (AFM) using an 5600LS atomic force microscope (Agilent Technologies, Inc., USA). CAT diagnostic reference measurements were conducted using a Jenway UV–visible spectrophotometer (Model 7205, Jenway Instruments, St Neots, UK).

### Fabrication of MIP/MWCNT/AgNP/GCE

Prior to the electropolymerization step, the GCE was polished using 0.3 μm alumina slurries to achieve a mirror-like surface appearance, placed in absolute ethanol and then in water for 5 min in an ultrasonic bath, and finally activated using CV runs in 0.5 M H_2_SO_4_.

Chronoamperometry was applied for the electrodeposition of AgNPs on the polished and activated GCE surface using a 0.1 M NaNO_3_ solution containing 5 mM AgNO_3_ for 300 s at a potential of −0.8 V [43]. Afterward, 4 μL of MWCNTs (1 mg/mL DMF) was drop-cast on the surface of the modified GCE and left to dry at room temperature. The MWCNT/DMF solution was sonicated prior to drop-casting on the electrode surface for at least 30 min to ensure the homogeneity of the solution and good dispersion of the particles in DMF, and to avoid any agglomeration.

The MIP was fabricated via voltammetric electropolymerization cycles of a solution composed of 5 mM *O*AP and 250 U/mL CAT prepared in PBS (0.05 M, pH ≈ 7) within a potential range of −0.2 V to 0.8 V for ten cycles at a scan rate of 50 mVs^−1^. The non-imprinted electrode (NIP/MWCNT/AgNP/GCE) was fabricated under the same conditions in the absence of CAT.

The morphology and topography of the bare GCE, AgNP/GCE, MWCNT/AgNP/GCE, MIP/MWCNT/AgNP/GCE, and NIP/MWCNT/AgNP/GCE surfaces were characterized using SEM and AFM techniques, and electrochemical analysis was performed using CV and EIS.

### Template elution

After electropolymerization, different solutions including Milli-Q water, PBS, Triton, methanol, 0.1 M H_2_SO_4_, methanol, and 0.1 M H_2_SO_4_ (4:1 v/v) were tested to determine the optimal solution for the elution of imprinted CAT from the fabricated polymeric matrix by immersing the modified electrode surface for different elution time intervals (1–10 min).

The applicable recognition and rebinding of the sensor to CAT was then investigated by incubation in PBS (pH ≈ 7.0) containing different concentrations of CAT (25, 50, 100, 150, 250, 300, 400, and 500 U/mL, respectively).

### Electrochemical measurements

Differential pulse voltammetry (DPV) measurements were carried out using a 250 U/mL CAT solution prepared in PBS (0.05 M, pH ≈ 7) at a potential range from −0.2 V to 0.8 V, amplitude of 10 mV, step potential of 5 mV, pulse width of 0.05 s, and pulse period of 0.1 s. CV measurements were performed at a potential range from −0.6 V to 0.9 V and a scan rate of 50 mVs^−1^. EIS experiments were performed at potential of 0.2 V, within a frequency range from 1 mHz to 100 kHz, and amplitude of 5 mV. All measurements were performed at room temperature using 2.5 mM [Fe (CN)_6_]^−3/−4^ solution (FCN) as the active probe.

### CAT detection in biological samples

The fabricated MIP/MWCNT/AgNP/GCE sensor was tested for CAT recognition in serum and urine samples. The urine analysis involved centrifugation of 10 mL of urine samples from healthy volunteers for 10 min at 10,000 rpm; the supernatant was then diluted 50 times in 0.05 M PBS (pH ≈ 7.0), without any additional treatment, and spiked with different CAT concentrations. Serum samples of anonymous volunteers were collected, and each 1 mL of serum was mixed with 2 mL of methanol and kept at −4 °C to precipitate serum protein. The mixture was then centrifuged for 10 min at 10,000 rpm, diluted 100 times in 0.05 M PBS (pH ≈ 7.0), and spiked with various CAT concentrations.

### Interference effect

The selectivity of the sensor was examined by measurement of CAT in the presence and absence of potential biological interfering compounds coexisting with CAT in plasma or urine. Stock solutions of glucose, urease, xanthine, creatine, uric acid, and CAT were prepared in 0.05 M PBS (pH ≈ 7.0). Appropriate volumes were mixed with CAT solution to obtain three different ratios of the prepared interferent solutions, at equimolar, fivefold, and tenfold interferent concentrations relative to 250 U/mL CAT (0.3 μM, calculated based on its labeled specific activity [2950 U/mg] and molecular weight [~240 kDa]), for electrochemical detection under optimal conditions.

## Results and discussion

### Optimization of the electrode surface prior to the imprinting process

**Fig. 1:**
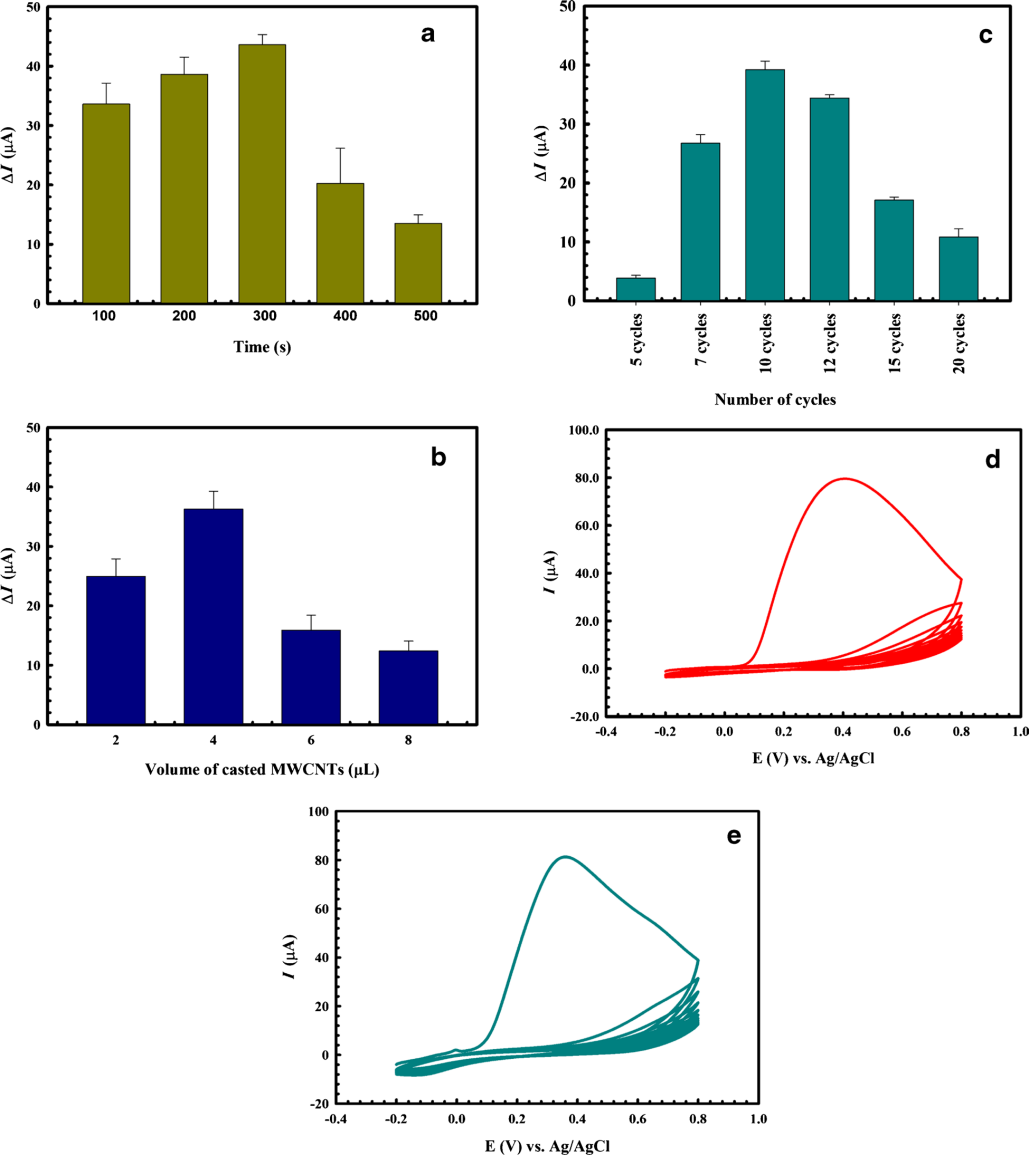
**a** Effect of silver deposition time, **b** MWCNT loading volume, and **c** number of cycles on the response of 2.5 mM FCN solution using DPV after incubation in 250 (U/mL) of CAT. Electropolymerization of *O*AP on the sensor surface versus Ag/AgCl by CV in a range from −0.2 V to 0.8 V in 0.05 M PBS for ten cycles at a scan rate of 50 mV s^−1^, in the **d** presence and **e** absence of 250 (U/mL) CAT. The error bars represent the standard deviation of three replications using the same sensor

The polished GCE was electrochemically activated using repetitive CV cycles in 0.5 M H_2_SO_4_ prior to chronoamperometric electrodeposition of AgNPs at various time intervals (100, 200, 300, 400, and 500 s), as shown in Fig. 1a, revealing that 300 s was the ideal deposition time [42].

To determine the effect of MWCNT loading volume, MWCNTs were drop-cast in different volumes (2, 4, 6, and 8 μL) on the modified AgNP/GCE surface, and as shown in Fig. 1b, 4 μL was found to be efficient for charge transfer enhancement. All optimization experiments were carried out using DPV, and the change in oxidation current (Δ*I*) of 2.5 mM FCN solution before and after each modification step was used to determine the optimal experimental condition.

### Preparation of the hybrid imprinted sensor

CAT was imprinted on the MWCNT/AgNP/GCE surface via electropolymerization of 5 mM *O*AP in the presence of 250 U/mL CAT prepared in PBS (0.05 M, pH ≈ 7), using CV in a range from −0.2 to 0.8 V in PBS (pH 7.0) for ten cycles at a potential scan rate of 50 mVs^−1^ as shown in Fig. 1c. The results showed that the greater the number of cycles, the greater the depletion in the *O*AP oxidation current, confirming the fabrication of the nonconducting polymeric film. Notably, no discernible change in the voltammogram of the electropolymerization was observed when CAT was involved, as shown in Fig. 1d (in the case of the imprinted sensor) or in its absence (Fig. 1e, in the case of the non-imprinted sensor), indicating that CAT did not undergo any electrochemical changes during the process of *O*AP electropolymerization [44].

### Optimization of experimental parameters

The performance of the engineered sensor may be affected by many factors, such as elution time and solvent, incubation time, number of electropolymerization cycles, and the ratio between template T and monomer M (T:M). Consequently, these parameters were studied in order to achieve maximum sensitivity for the developed sensor.

The optimal number of CV scan cycles during the electropolymerization process was examined by testing its effect on the response of 2.5 mM FCN active probe after incubation of the modified sensor in 250 U/mL CAT solution following treatment with varying numbers of electropolymerization cycles (5, 7, 10, 12, 15, and 20 cycles) in order to improve the sensor’s sensitivity and stability. Figure 1c shows that the Δ*I* value increased up to ten cycles, after which it decreased as the number of cycles increased.

This can be explained as follows. As the number of cycles increases, the imprinted polymeric film may become thicker, allowing CAT molecules to be entrapped in its recognition cavities and preventing their effective removal from the polymeric matrix. This may result in a blocked cavity that does not alter the FCN response or in template leakage in subsequent experiments. On the other hand, an insufficient number of scan cycles may result in the formation of a very thin imprinted polymeric film that can be easily leached from the sensor surface during the washing steps, thereby increasing nonspecific adsorption and decreasing the number of CAT recognition cavities on the polymeric matrix [32, 45].

**Fig. 2:**
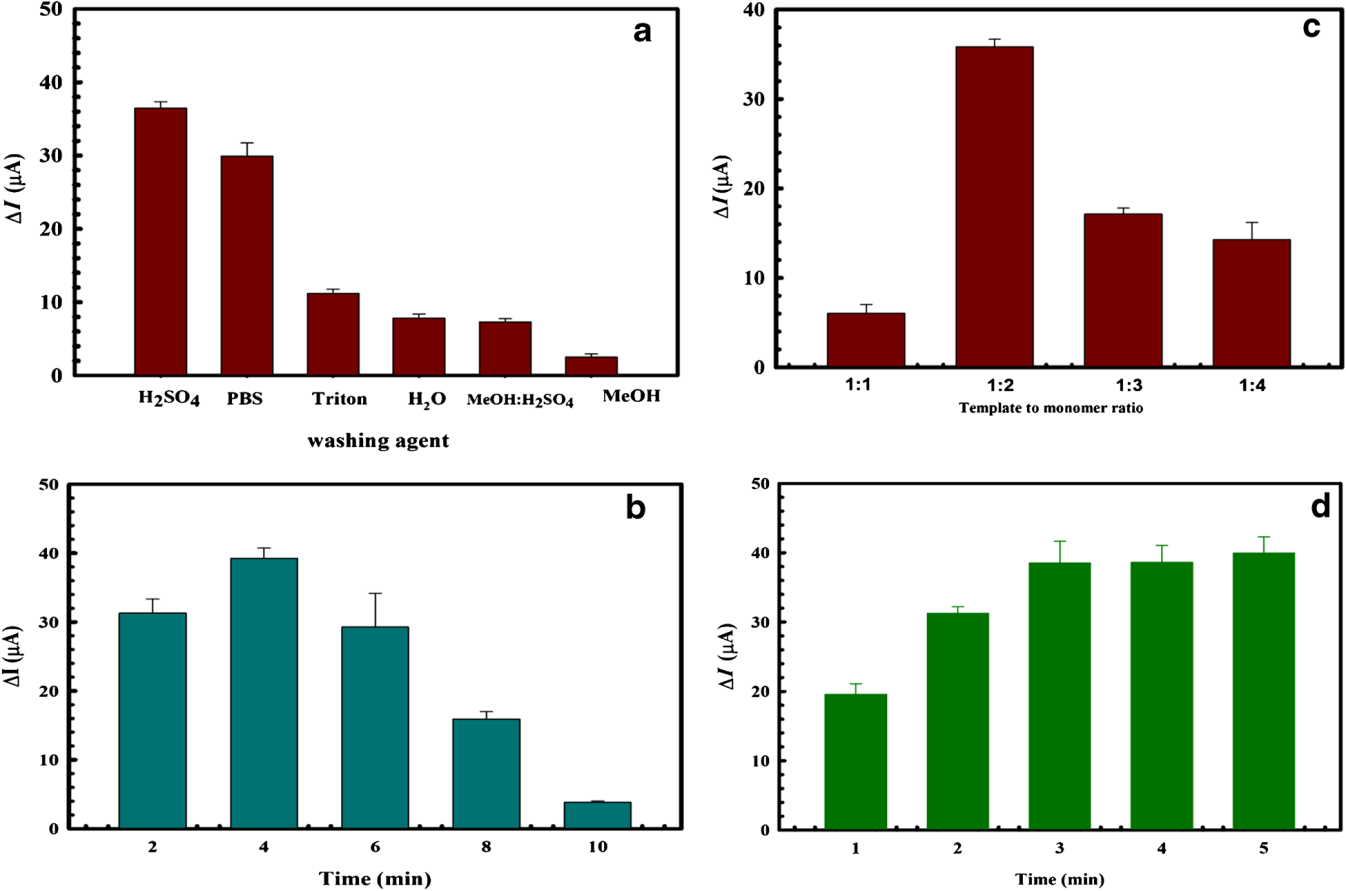
Effect of **a** elution solvents, **b** elution times, **c** template/monomer ratio, and **d** incubation time on the response of 2.5 mM FCN solution using DPV after incubation in 250 U/mL CAT. The error bars represent the standard deviation of three replications using the same sensor

CAT elution from the imprinted matrix was tested using a variety of solvents, including Milli-Q water, PBS, Triton, methanol, 0.1 M H_2_SO_4_, and a mixture (4:1 v/v) of methanol and 0.1 M H_2_SO_4_. The Δ*I* values for DPV measurements of 2.5 mM FCN solution were determined by the change in recorded values from before to after soaking in the various extraction solvents. As shown in Fig. 2a, immersing the electrode in 0.1 M H_2_SO_4_ resulted in the highest Δ*I*; accordingly, it was regarded as the most efficient solvent for CAT removal.

The optimal time for soaking the proposed sensor in the elution solvent, 0.1 M H_2_SO_4_, was also investigated by testing different time intervals (2, 4, 6, 8, and 10 min). Figure 2b indicates that 4 min was sufficient for the elution process. Increasing the washing time might cause a reversible interaction and rebinding of CAT with its recognition cavities, thus reducing the recorded Δ*I* value [45].

The T:M ratio used in the electropolymerization step was tested at several ratios (1:1, 1:2, 1:3, and 1:4), as indicated in Fig. 2c. The results clearly show that the ratio of 1:2 is optimal, exhibiting the highest Δ*I*. The decrease in Δ*I* with the use of other ratios indicates that a lower ratio may produce fewer binding sites, whereas an additional increase in *O*AP might lead to a decrease in the specific binding to CAT, an increase in nonselective binding sites, and a thicker polymeric layer that could make it more difficult to extract the template [46].

The incubation time needed for the rebinding of CAT to its recognition sites of the sensor was optimized at different time intervals of 1, 2, 3, 4, and 5 min of soaking in 250 U/mL CAT, as shown in Fig. 2d. It is clear that 3 min was sufficient, as revealed from the highest Δ*I* value achieved. This fast rebinding to the recognition sites resulted in the fast response of the proposed sensor, which is considered a key advantage of the application of electrochemical sensors compared with traditional methods of analysis [46].

### Surface characterization of the fabricated sensor

#### AFM characterization for different fabricated surfaces

**Fig. 3:**
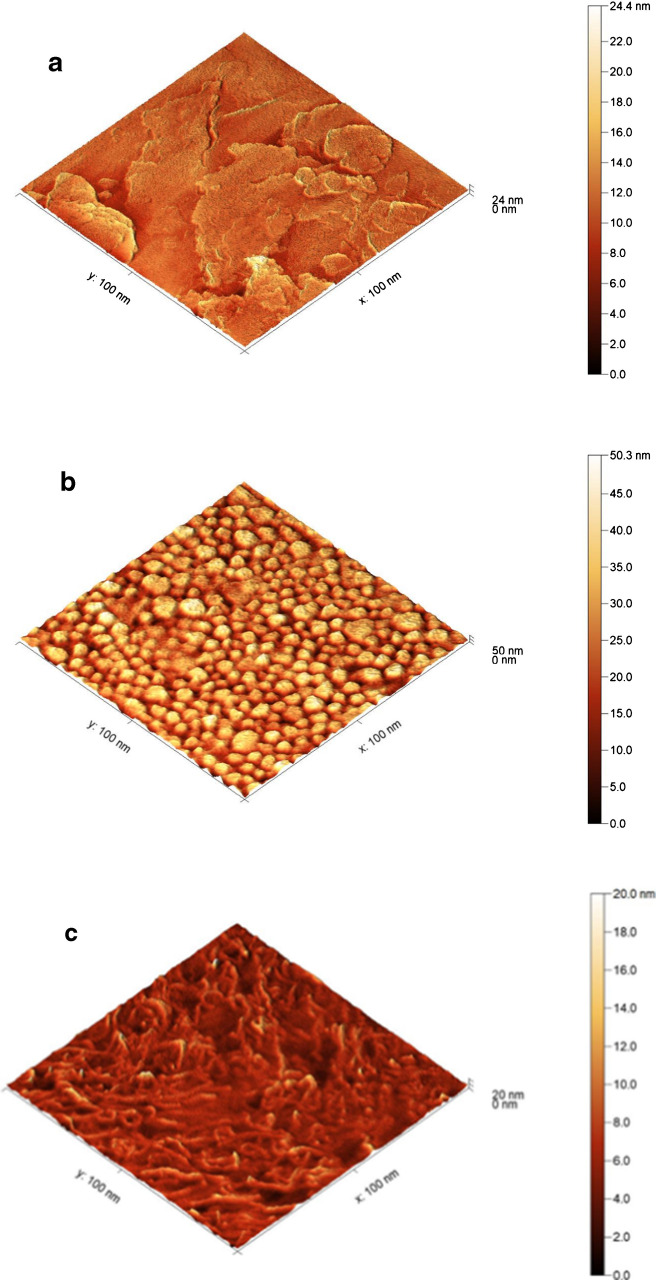

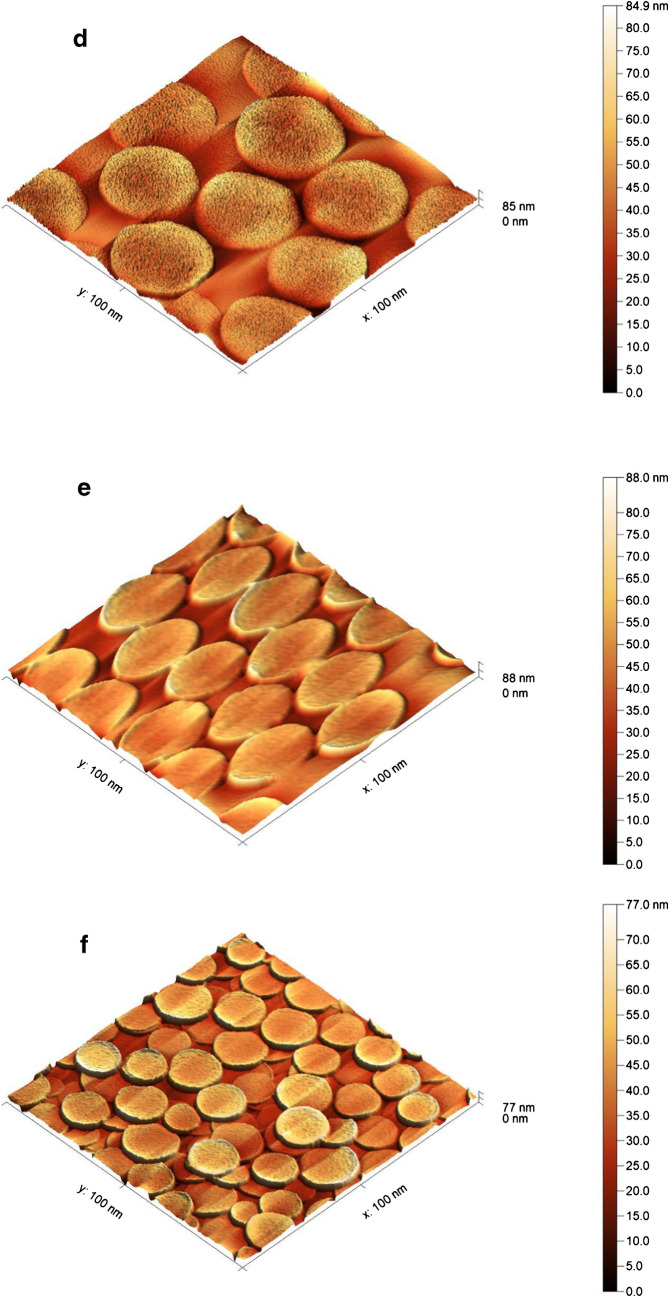
Two-dimensional surface height topography images using AFM: **a** bare GCE, **b** GCE modified with AgNPs, **c** GCE modified with MWCNTs. **d** MIP with CAT, **e** CAT-free MIP, **f** NIP

The topography, surface height profiles, and root-mean-square (RMS) roughness of the fabricated sensor were determined by AFM characterization, as shown in Fig. 3. Different surface modification steps were performed on the GC tips (diameter = 3 mm, length = 10 mm) directly connected to the potentiostat using a crocodile clip as a connector, and the other axial area was completely isolated using a Teflon ribbon.

An RMS roughness value of 1.94 nm was recorded for the bare GCE surface, as shown in Fig. 3a. After the surface modification with AgNPs, the RMS was increased to 7.42 nm (Fig. 3b). On the other hand, MWCNT/AgNP/GCE surface fabrication produced an RMS value of 1.59 nm, as indicated in Fig. 3c. The recorded difference in the RMS at the different steps indicates the formation of the several surfaces of the modifiers. The regular MIP-modified surface in Fig. 3d showed an RMS value of 10.07 nm, and after the washing step using 0.1 M H_2_SO_4_ solution, the RMS value was increased to 10.73 nm, which confirms the successful removal of CAT from the fabricated MIP sensor and the formation of the cavities (Fig. 3e). The RMS value for the NIP film (prepared via electropolymerization in the absence of CAT) shown in Fig. 3f was 10.90 nm, which indicates the successful fabrication of the irregular NIP polymeric film in the absence of the template.

The variation in the recorded roughness of the non-imprinted film relative to the imprinted films can be attributed to the irregularity and polymer chain enlargement in the absence of template molecules. These results were found to agree with previously reported AFM studies on the morphological characterization of imprinted sensors using electropolymerization [47, 48].

#### SEM characterization for different fabricated surfaces of the sensor

**Fig. 4:**
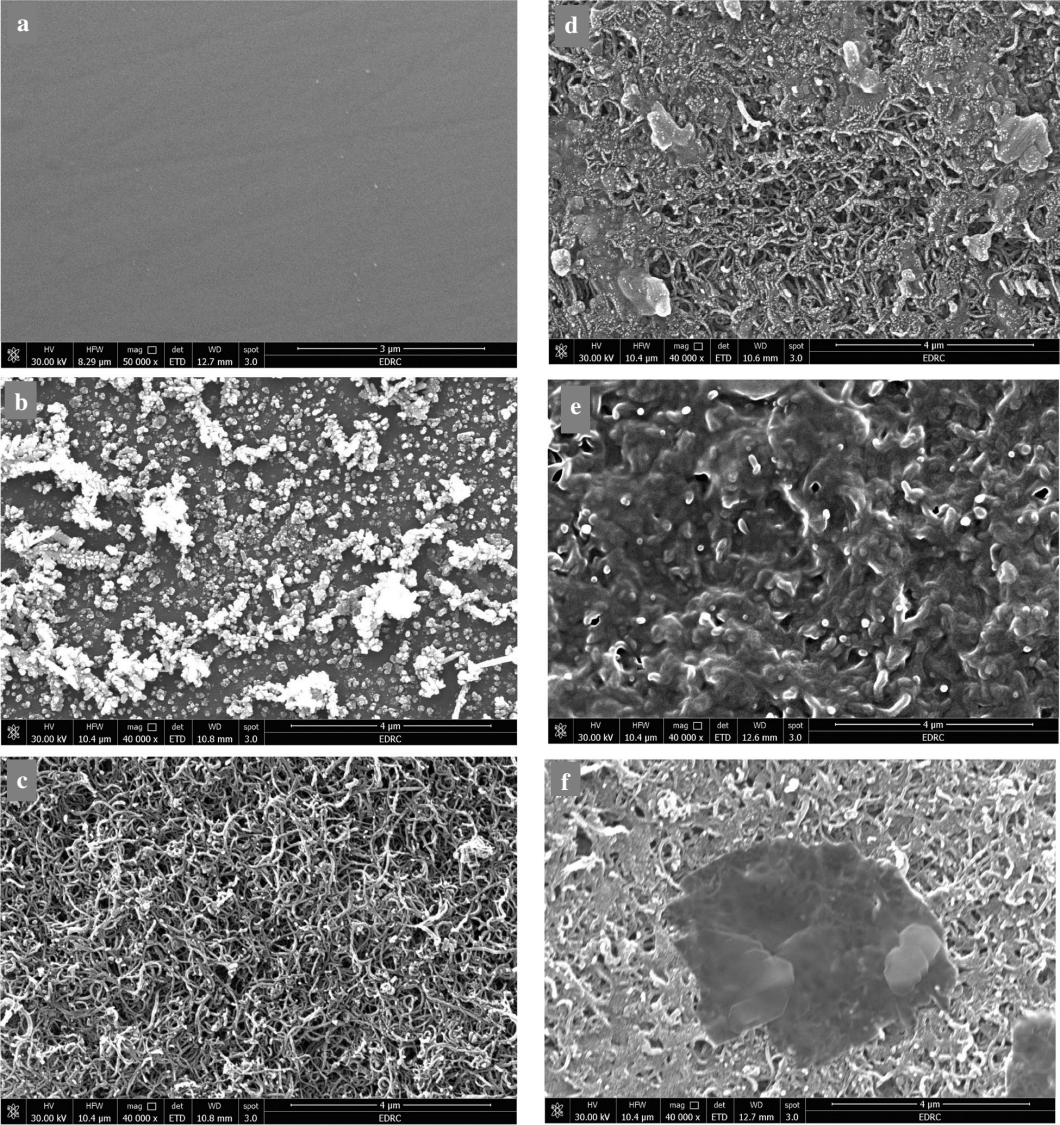
SEM images showing the topography of the proposed sensor surface of **a** bare GCE, **b** GCE modified with AgNPs, **c** GCE modified with MWCNTs, **d** MIP with CAT, **e** CAT-free MIP, **f** NIP

SEM characterization was utilized to describe the various surface alterations that resulted from each modification procedure, beginning with the electrodeposition of the AgNPs, followed by the casting of MWCNTs, the CAT enzyme imprinting into the *O*AP polymer matrix, and its removal from the MIP matrix, as well as for the NIP, as shown in Fig. 4.

The spherical shape of the AgNPs uniformly deposited onto the GCE surface is evident in Fig. 4b, in contrast to Fig. 4a, which displays the bare electrode surface. The diameters of randomly selected nanoparticles, excluding agglomerated clusters, were determined using ImageJ software after setting the scale using the scale bar. The calculated average particle size was 16.7 ± 11.0 nm, indicating a relatively narrow size distribution for electrochemically deposited AgNPs.

The fabrication of the second modified layer using the drop-cast MWCNTs was clearly confirmed by the tube-like shapes, indicating the efficient and uniform deposition of MWCNTs, as indicated in Fig. 4c.

With a different morphology, irregular structure, and pore appearance following the CAT removal process, the cavities resulting from the knockout of the CAT template from the MIP-modified surface can be verified by comparing Fig. 4d and e, which show the MIP-modified GCE before and after the CAT elution, respectively.

The NIP (Fig. 4f), on the other hand, exhibits a homogeneous morphology without notable porosity as a result of the absence of a template during polymerization. The MIP- and NIP-modified surfaces display significant morphological differences, which validates the successful imprinting process and the development of specific recognition sites that are compatible with the target molecules.

### Electrochemical characterization

CV and EIS were applied to characterize the step-by-step fabrication of the sensor using 2.5 mM FCN as the electroactive probe.

**Fig. 5:**
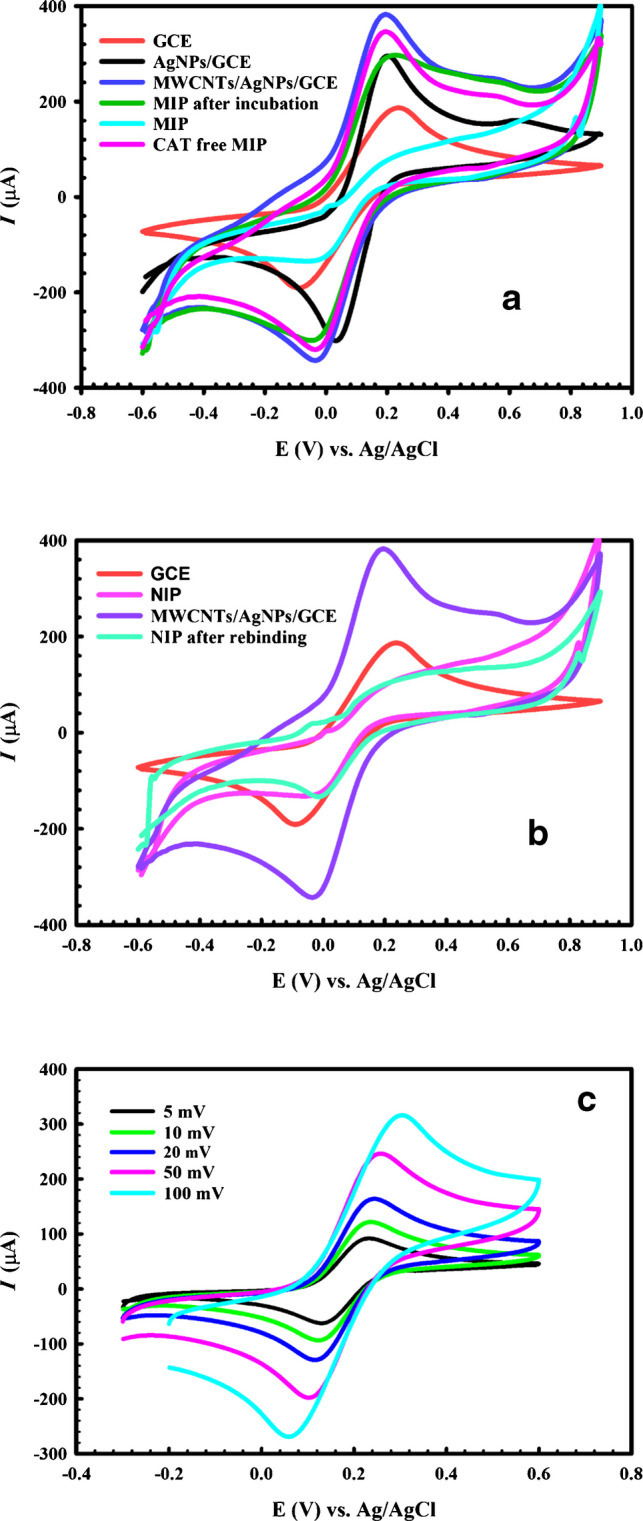

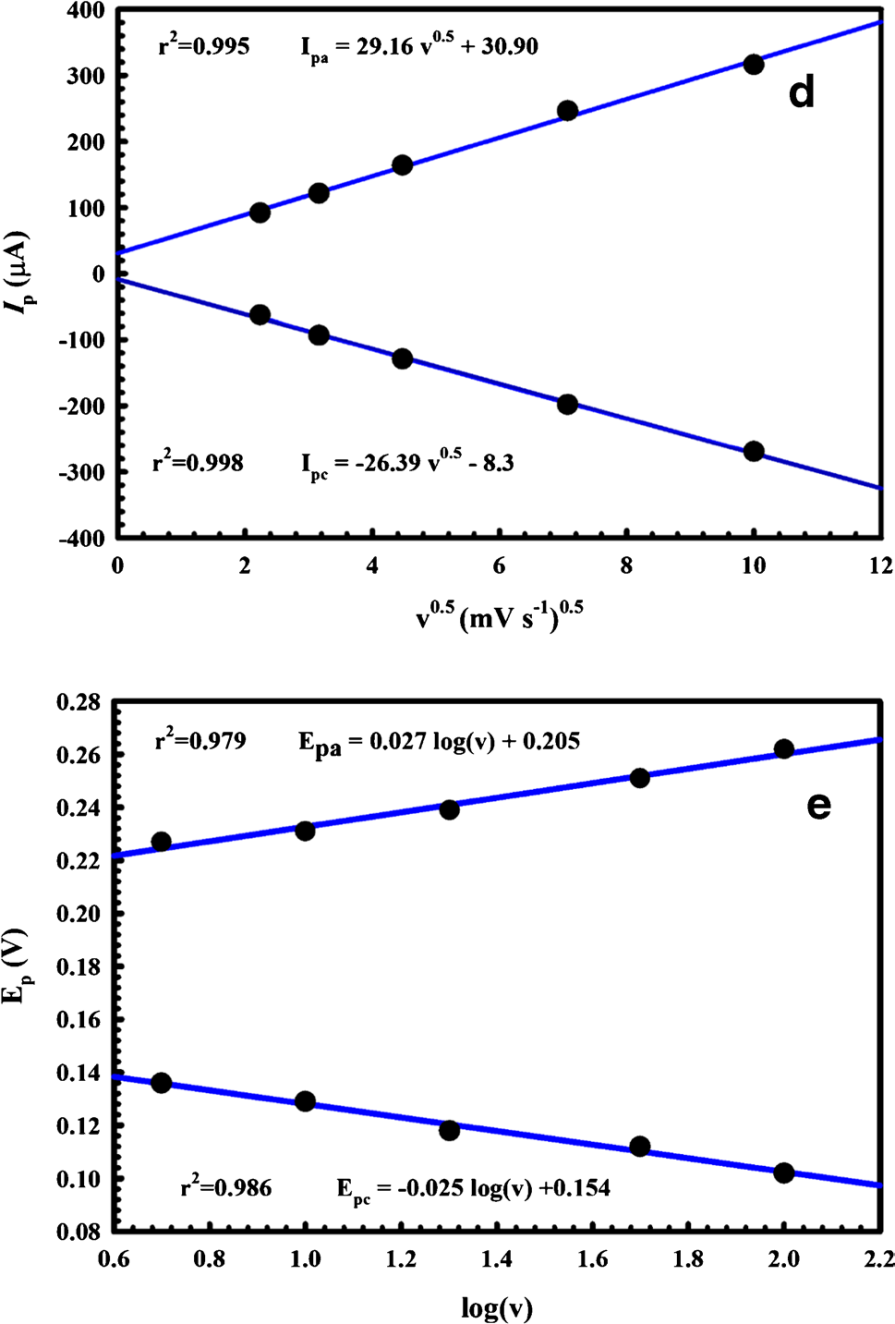
**a** CVs of the stepwise fabrication of the proposed MIP sensor versus Ag/AgCl at a potential scan rate of 50 mV s^−1^ in the potential range of −0.6 V to 0.9 V. **b** CVs of the stepwise fabrication of the NIP sensor at a scan rate of 50 mV s^−1^ in a potential range of −0.6 V to 0.9 V. **c** CVs of 2.5 mM FCN for the proposed sensor at different scan rates. **d** Variation in cathodic and anodic peak current with the square root of the scan rate (*n* = 3). **e** Variation in cathodic and anodic peak potential with the logarithm of the scan rate

The CV of the bare GCE indicates two distinct redox peaks corresponding to the FCN reversible electron transfer process (Fig. 5a, bare GCE). After AgNP electrodeposition, the current was found to increase as a consequence of the increased electroactive surface area (Fig. 5a, AgNP/GCE), and the optimal current increase was achieved after the drop-casting of MWCNTs on the AgNP/GCE surface (Fig. 5a, MWCNT/AgNP/GCE).

Moreover, the decrease in the redox peaks upon electropolymerization of the CAT imprinted layer (Fig. 5a, MIP) can be attributed to the nonconductive nature of the *O*AP polymeric membrane that resulted in the blocking of the electrode surface.

Upon CAT extraction using 0.1 M H_2_SO_4_ to create the washed MIP surface, the cavities formed enhanced the FCN passage to the substrate, which resulted in an increase in the redox peak current (Fig. 5a, CAT-free MIP). After 3 min of soaking the washed MIP sensor in 250 U/mL CAT solution, the CAT molecules were capable of rebinding to their cavities, which had the same shape and size as CAT, as confirmed by the significant decrease in current, indicated by the *ΔI* value (Fig. 5a, MIP after CAT rebinding).

It should be noted that the reduction in the redox current upon testing of the NIP sensor shown in Fig. 5b can be attributed to the nonconductive film fabrication, which prevented the FCN probe from reaching the substrate surface. Taking into consideration that the NIP polymeric film has no imprinted cavities, the current difference between the NIP surface before and after soaking in 250 U/mL CAT in Fig. 4b seemed to be unremarkable, indicating the absence of nonspecific CAT adsorption on the surface.

The electrochemical mechanism was further investigated by measuring the cyclic voltammograms of 2.5 mM FCN solution for the MIP after the washing step at various potential scan rates ranging from 5 to 100 mVs^−1^, as illustrated in Fig. 5c. A linear relationship of the anodic peak current (*Ip*_a_) and cathodic peak current (*Ip*_c_) with the square root of the potential scan rate (v^1/2^) was identified (Fig. 5d), with the corresponding regression formulas as follows:

$$\begin{array}{cc}I{p}_{c}\left(\mu A\right)=-8.30-26.39{v}^{1/2}& ({R}^{2}=0.998)\\ I{p}_{a}\left(\mu A\right)=30.90+29.16{v}^{1/2}& \left({R}^{2}=0.995\right)\end{array}$$

Figure 4e shows the linear relationship between the anodic and cathodic peak potentials and the logarithm of the scan rate; thus, the FCN reaction on the MIP electrode can be regarded as a diffusion-controlled process.

The Randles–Sevcik equation was also applied, and the slope of the correlation between the anodic peak current and the square root of the scan rate was used to calculate the active surface area of the sensor as follows [49]:

$${\mathrm{Ip}}_{\mathrm{a}}=\left(2.69\times {10}^{5}\right){\mathrm{n}}^{3/2}{\mathrm{A}}{\left({\mathrm{D}}_{\mathrm{o}}\right)}^{1/2}{\mathrm{C}}_{\mathrm{o}}{{\mathrm{v}}}^{1/2},$$

where *n* is the number of electrons transferred for FCN (*n* = 1), *A* is the electrode surface area, *D*_0_ is the diffusion coefficient of FCN = 7.6 × 10^−6^ cm^2^ s^−1^, and C_0_ is the probe concentration (2.5 mM). An effective surface area of 0.498 cm^2^was found for the modified MIP/MWCNT/AgNP/GCE sensor, which is roughly seven times the bare surface area (0.071 cm^2^).

**Fig. 6:**
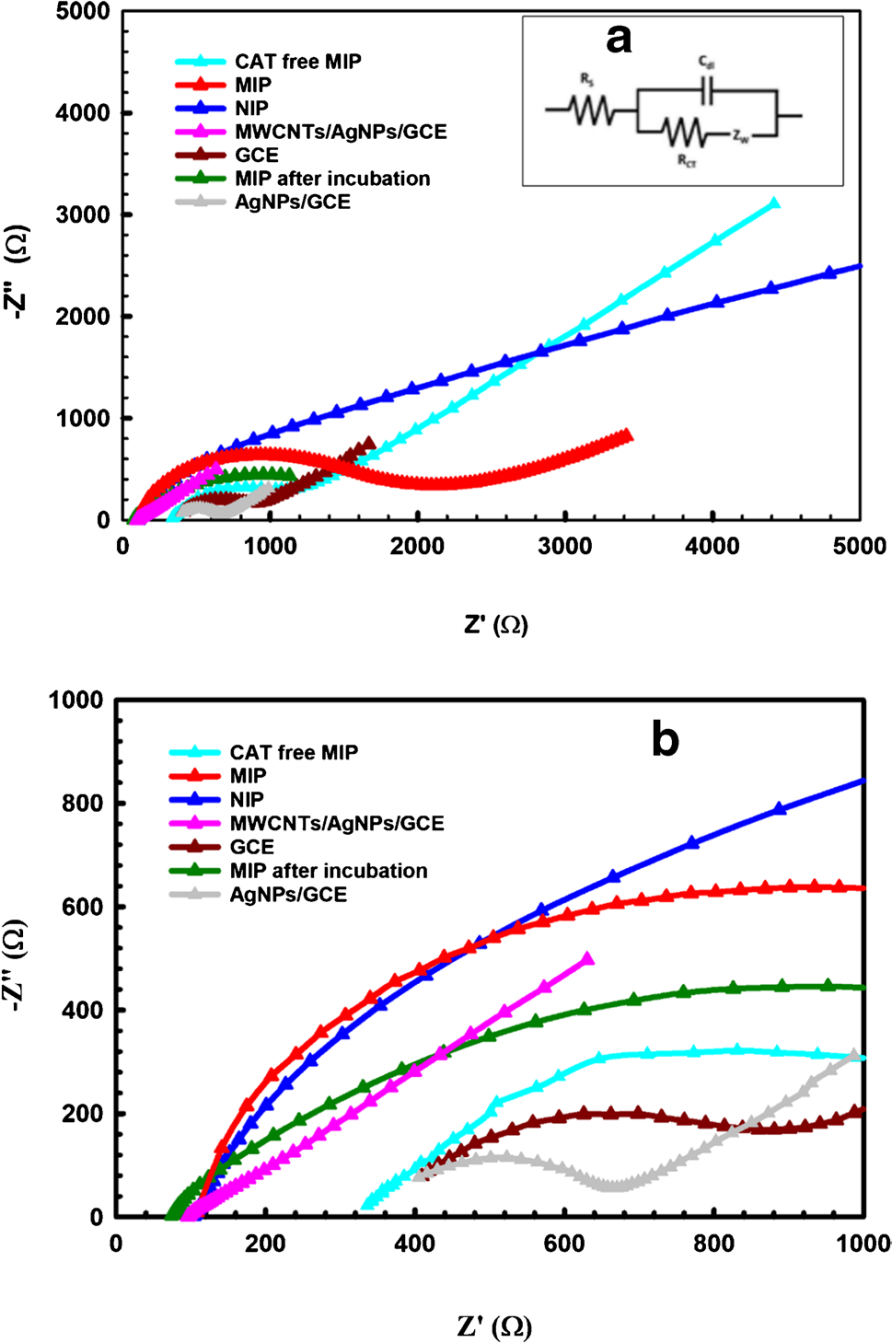
EIS of the stepwise fabrication of the MIP/MWCNT/AgNP/GCE-modified MIP sensor at potential of 0.2 V, frequency range of 0.1 Hz to 100 kHz, and amplitude of 0.01 V. **a** Randles equivalent circuit of the fitted EIS data, and **b** zoomed-in region of high frequency

EIS is considered a reliable tool for studying the sensor surface interface properties [50]. Nyquist plots of the sensor’s stepwise fabrication are shown in Fig. 6; each plot consists of two sections: a semicircle at the high-frequency region, and a lower frequency that shows a linear component, with the charge transfer resistance (*R*_ct_) equal to the semicircle diameter. The resistance can be estimated by fitting the EIS data to the Randles equivalent circuit (Fig. 6a inset).

The *R*_ct_ for the AgNP/GCE sensor was 243.0 Ω, and the recorded *R*_ct_ for AgNP/MWCNT/GCE was 21.0 Ω, which is significantly less than that of the bare GCE, at 421.3 Ω. This indicates that the rate of electron transfer significantly increased following the surface modification steps. With the further development of nonconductive *O*AP polymeric films on the substrate surface, *R*_ct_ values of 884.6 Ω and 1636.0 Ω were recorded for NIP and MIP, respectively, as displayed in Fig. 6.

In contrast to the compact and nonspecific NIP layer, the MIP layer exhibits specific recognition cavities that act as an insulating layer and increase charge transfer resistance. This is the reason for the notable difference between the MIP and NIP EIS spectra.

However, when CAT was extracted from the polymeric matrix, the *R*_ct_ value dropped due to the regeneration of free recognition cavities on the film, resulting in a notable increase in the rate of electron transfer (Fig. 6, MIP CAT-free). The *R*_ct_ value increased once again when the sensor was soaked in a CAT solution, which is attributed to the rebinding of the CAT molecules to the film’s recognition cavities, blocking electron transfer between the substrate and the redox probe as a result of the imprinting effect.

### Electrochemical performance of the proposed sensor

#### CAT detection using the MIP/MWCNT/AgNP/GCE sensor

**Fig. 7:**
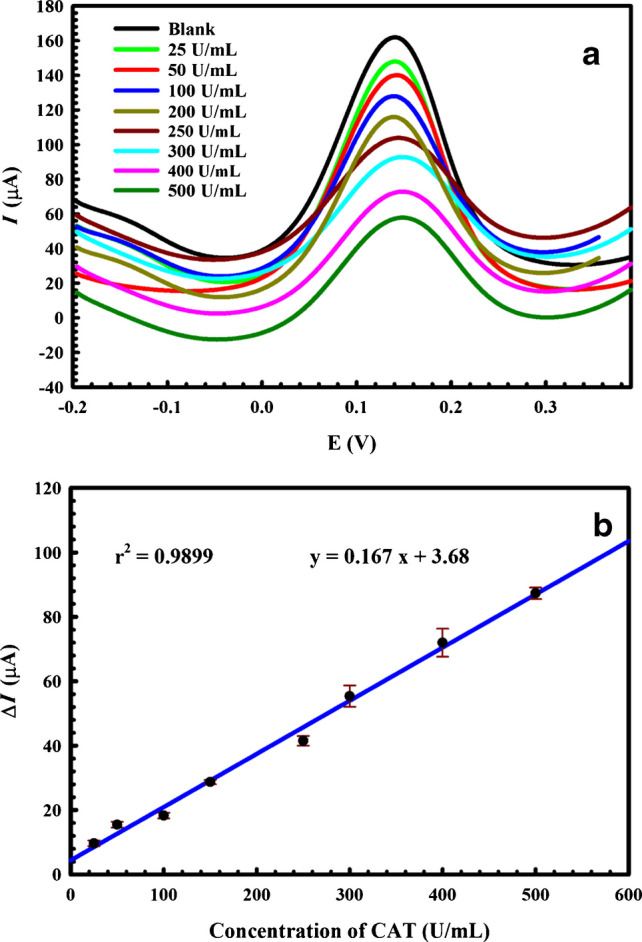
**a** DPV peaks of FCN of the MIP sensor after soaking in different concentrations of CAT. **b** Corresponding calibration curve of the sensor. Error bars represent the standard deviation of three replicates

DPV responses of 2.5 mM FCN in PBS (pH 7.0) were recorded under optimal experimental conditions after soaking the electrode in a range of CAT concentrations from 25 to 500 U/mL to confirm that the proposed MIP electrode is suitable for CAT sensing. As shown in Fig. 7, the redox probe current decreases as the CAT concentration increases because of the increased occupancy of the cavities by CAT molecules, which in turn reduces the available sites for the interaction of FCN, the active probe, thereby reducing its acquired signal.

A linear relationship was recorded over the range of 25–500 U/mL (R = 0.993, *n* = 3) for the *ΔI* response of the active probe, before and after incubation, which can be represented by the following equation:

$${\triangle}I =0.156\left[{\mathrm{CAT}}\right]\left({\mathrm{U}}/{\mathrm{mL}}\right)+4.397.$$

In addition, by comparing the measured *ΔI* of low concentrations of CAT with the *ΔI* of the blank sample (PBS, pH 7), the limit of detection (LOD), or the lowest amount of CAT in the sample that can be detected, is 3.59 U/mL, but not necessarily quantified as an exact value, based on a signal-to-noise ratio (S/N) = 3 [51].

#### MIP/MWCNT/AgNP/GCE sensor selectivity

**Fig. 8:**
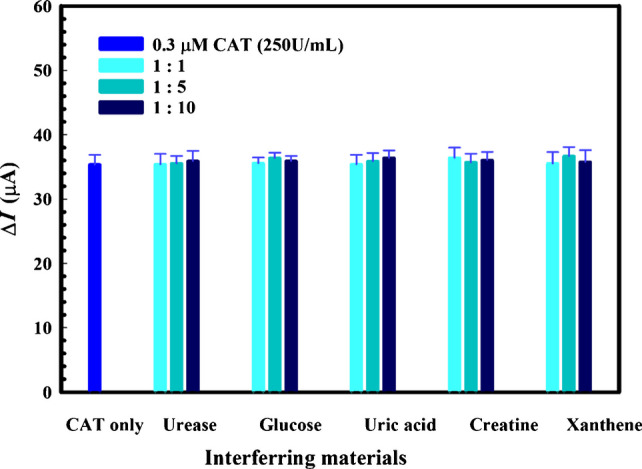
Selectivity of the sensor toward 0.3 μM CAT (250 U/mL) in the presence and absence of the tested interfering materials. The error bars represent the standard deviation of three replicates using the same sensor

The selectivity of the proposed MIP/MWCNT/AgNP/GCE sensor toward the CAT enzyme was evaluated at various ratios of other potential species present in biological fluids. DPV was applied to evaluate the rebinding and response of the imprinted sensor after knocking out the template following its incubation in different solutions comprising a fixed concentration of CAT 0.3 μM (250 U/mL) and concentrations of glucose, uric acid, creatine, xanthine, or urease that were equal to, five times, and ten times that of CAT, as shown in Fig. 8.

Even at high concentrations, the presence of these interfering species did not significantly affect the determination of CAT by the imprinted sensor, as demonstrated by the results displayed in Fig. 8, where no significant change in the *ΔI* values was observed at the tested interference levels, and CAT recovery percentages in the range of 100.1–103.7% were achieved despite the presence of high concentrations of these species. This confirms the high selectivity of the imprinted cavities as CAT recognition sites.

### Analytical applications and validation

The MIP/MWCNT/AgNP/GCE sensor was applied for the detection of CAT in spiked urine and serum samples obtained from anonymous volunteers. A calorimetric kit (BIO Diagnostics and Research Reagents Ultimate CAT Kit, Cairo, Egypt) was used to determine CAT concentration as a reference method. The proposed sensor was utilized for CAT detection in real samples, using the standard addition method at different spiked standard concentrations of CAT (15, 25, 50, 100, and 150 U/mL) with real samples of serum and urine, and the recoveries obtained were compared with the reference kit values. The results as presented in Table 1 confirm the accuracy of the MIP/MWCNT/AgNP/GCE sensor, with recovery percentages ranging from 95.73% to 101.60% and relative standard deviation (RSD) values between 0.41% and 1.52%, and with good agreement between the results obtained and those of the commercial kit.

**Table 1:** Application of the proposed MIP/MWCNT/AgNP/GCE sensor to real samples in comparison with the reference kit colorimetric method

Sample	Spiked amount^a^ (U/mL)	Found (U/mL)	Recovery (%)	RSD^b^ (%)
Serum	15	14.62	97.46	1.02
	25	25.07	100.28	0.84
	50	50.22	100.44	0.86
	100	100.24	100.24	0.63
	150	150.16	100.11	0.41
Urine	15	14.36	95.73	1.18
	25	25.40	101.60	1.10
	50	50.56	101.12	0.67
	100	99.82	99.82	0.18
	150	150.08	100.05	1.52

^a^Spiked amount = the spiked CAT amount measured by the reference colorimetric kit

^b^RSD = relative standard deviation (*n* = 3) using the same electrode

The proposed sensor was validated according to the International Conference on Harmonisation (ICH) guidelines [52] in terms of its linearity, selectivity, LOD, repeatability, and reproducibility. The method was applied in a linear range of 25–500 U/mL, demonstrating a LOD of 3.59 U/mL, high selectivity toward CAT, and good reproducibility and stability, as shown by RSD values of 0.41–1.52%. The ability of an analytical approach to produce identical results under various conditions, such as varying one of the materials used, the devices, the personnel, or the period of measurement and/or location, is known as reproducibility [53].

Three different MIP sensors were tested by measuring the change in current and calculating the RSD values, which were found to be 2.60%, indicating good reproducibility. However, by using a single sensor to measure a single CAT concentration five times in a row (on the same day, *n* = 5), repeatability, which can be defined as the capacity to achieve the same results using a particular analytical method under the same conditions of measurement, was examined, and the achieved RSD of 1.54% indicated the sensor’s acceptable repeatability.

The MIP sensor was stored in a refrigerator for 30 days at 4 °C to test its shelf stability. The stability of the sensor was determined by recording the current response to 2.5 mM FCN after exposure of the sensor to a CAT solution at a concentration of 250 U/mL, and it was found to retain 97.3%, 90.6%, and 86.9% of its initial value after 10, 20, and 30 days, respectively, indicating its high stability.

The proposed sensor demonstrates higher sensitivity in terms of detection limit when compared with previously published electrochemical and spectrophotometric techniques, as shown in Table 2. However, the main value of the current work lies in the fact that the method does not depend on the enzyme activity but rather on the concentration of CAT molecules; thus, the sensor maintains its structure and surface characteristics, resulting in generally higher reusability (polymer stability) than the other recently reported techniques, which are restricted by the stability and activity of the enzyme toward H_2_O_2_ as a substrate. Given the difficulty in developing noninvasive medical point-of-care testing instruments that deliver accurate results and less invasiveness and discomfort for patients during sampling, the proposed method is an excellent approach for CAT detection, with advantages of affordability, robustness, and ease of accessibility.

**Table 2:** Analytical parameters for CAT determination using other recently reported methods in comparison with the current work

Method	Linear range	LOD	Ref
Electrochemical MIP/MWCNT/AgNP/GCE sensor	25–500 U mL^−1^	3.59 U mL^−1^	This work
Catalase electrified liquid–organogel interface	1–20 μM	0.42 μM	[54]
MnO_2_-modified potentiometric electrode for catalase activity (via H_2_O_2_)	0.16–3.26 M	11–23 U mL^−1^	[55]
Electrochemical membrane-based pressure sensor (MePS)	396 pM–100 nM	396 pM	[56]
Catalase (Fe@G-MWCNTs) screen-printed electrode	0.1–7 mM	28.2 μM	[57]
Kinetic method	0.10–5 U mL^−1^	**–**	[58]
Chromogenic method	0.32–10 U mL^−1^ 1.25–105.52 μmol L^−1^	0.12 μmol L^−1^	[19]

## Conclusion

This work presents the first CAT hybrid sensor based on AgNPs and MWCNTs as response enhancers and *O*AP electropolymerized imprinted polymer as a molecular recognition element for CAT rather than reactivity toward its substrate. CAT was successfully eluted from the MIP using 0.1 M H_2_SO_4_, creating complementary cavities equivalent in shape and size to the studied template (CAT). These formed cavities allowed the FCN probe to pass, leading to the appearance of the redox peak. The sensor showed linearity from 25 to 500 U/mL, with a LOD of 3.59 U/mL (S/N = 3). The proposed MIP/MWCNT/AgNP/GCE sensor can be applied without any complicated preparation procedures and with a very short response time, using a portable device, making it suitable for point-of-care clinical field diagnostics and monitoring of CAT levels in clinical patients.

## Data Availability

Data and related materials will be available upon request from the corresponding author.
